# Clinical Outcomes of Penicillin‐Based Therapy Compared to Alternative Enterococcal Treatment in Patients With Vancomycin‐Susceptible Enterococcal Bloodstream Infections: A Monocentric Cohort Study

**DOI:** 10.1155/tbed/3822590

**Published:** 2026-08-27

**Authors:** Thalia Landrock, Lisa Schneider, Stefan Glöckner, Stefan Hagel, Mathias W. Pletz, Christina Bahrs

**Affiliations:** ^1^ Institute of Infectious Diseases and Infection Control, Jena University Hospital/Friedrich-Schiller-University, Jena, Germany, uni-jena.de; ^2^ Division of Infectious Diseases, Department of Internal Medicine I, Medical University of Vienna, Vienna, Austria, meduniwien.ac.at; ^3^ Institute of Medical Microbiology, Jena University Hospital/Friedrich Schiller University, Jena, Germany, uni-jena.de

**Keywords:** 30-day mortality, aminopenicillin, bacteremia, *Enterococcus faecalis*, *Enterococcus faecium*, septic shock

## Abstract

The aim of this retrospective cohort study was to evaluate clinical outcomes of penicillin‐based therapies (ampicillin, ampicillin/sulbactam, or piperacillin/tazobactam) compared with alternative therapies (glycopeptides, linezolid, or daptomycin) in patients with vancomycin‐susceptible enterococcal bloodstream infections (BSIs). Adult patients with a positive blood culture (BC) for a vancomycin‐susceptible *Enterococcus* species were screened from January 2017 to December 2018. Patients who received adequate antibiotic therapy for at least 48 h were enrolled. The primary outcome, 30‐day mortality, was compared between patients receiving penicillin‐based and those receiving alternative therapies using the Fisher exact test. Secondary outcomes included 90‐day mortality, relapse BSI, length of hospital stay (LOS), and potential antibiotic‐related adverse events. To identify independent predictors of 30‐day mortality, multivariable logistic regression analysis was weighted for ampicillin susceptibility using propensity scores. Of the 150 patients screened, 127 (84.6%) were included in the final analysis. Among them, 36 (28.3%) developed a septic shock. Aminopenicillin‐susceptible *Enterococcus* species (mainly *E. faecalis*) accounted for 59.1% of bacteremia cases. Sixty‐seven (52.1%) patients received a penicillin‐based regimen (monotherapy *n* = 48, combination *n* = 19), and 60 (47.2%) patients received an alternative regimen. Patients receiving penicillin‐based therapy exhibited a lower 30‐day mortality rate of 13.4% compared to 40% (*p* = 0.001) in those receiving alternative treatments. The 90‐day mortality rates were reduced (13.4% versus 41.7%), and LOS was shorter (median 21 versus 35 days) for patients with penicillin‐based therapy. There was no significant difference in relapse BSI or potential adverse events between the groups (*p*  > 0.05). However, in the multivariable weighted regression model, only septic shock (aOR with 95% CI: 23.53 [1.66; 334.54]) was identified as an independent positive predictor of 30‐day mortality. In conclusion, penicillin‐based therapy was still an effective and safe treatment but was not associated with an improved survival in a weighted analysis of patients with enterococcal BSI.

**Summary**

Penicillin‐based therapy was an effective and safe treatment in patients with enterococcal bloodstream infection (BSI) but was not associated with improved survival after propensity score weighting for aminopenicillin susceptibility.

## 1. Introduction

Enterococci are Gram‐positive coccoid‐shaped bacteria that reside in the intestines of healthy humans and animals [1]. However, they can cause various systemic infections in hospital settings and are common pathogens in bloodstream infections (BSIs) [2].

Enterococcal BSI is associated with high morbidity and mortality, particularly in patients with severe underlying conditions [3–5]. A systematic review and meta‐analysis from the WHO European Region, encompassing epidemiological studies between 2010 and 2020, reported all‐cause mortality rates for enterococcal BSI ranging from 14.3% to 32.3% [2]. Similarly, a recently published prospective multicenter cohort study from Spain found in‐hospital mortality rates of 20.8% for monomicrobial enterococcal BSI [6].

Among the numerous members within the genus, *Enterococcus faecalis* and *Enterococcus faecium* are most frequently associated with enterococcal BSI [5, 7, 8]. This trend is corroborated by data from the Australian Enterococcal Surveillance Outcome Program of 2023, which reported that out of 1599 enterococcal BSIs, 51.8% were attributed to *E. faecalis* and 41.1% to *E. faecium* [9]. These two primary species differ in their in vitro susceptibility to aminopenicillins. Recent population‐based surveillance data from the EU/EEA highlight the epidemiological significance of these pathogens: in 2023, over 92% of invasive *E. faecium* isolates tested in Europe were resistant to aminopenicillins, whereas *E. faecalis* largely remained susceptible. This notable resistance pattern has significant therapeutic implications and underscores the importance of ongoing local and international surveillance [10]. For invasive enterococcal infections caused by aminopenicillin‐sensitive pathogens such as *E. faecalis*, a penicillin‐based regimen, is the treatment of choice [4]. However, for *E. faecium*, alternative treatment options, such as glycopeptides, are warranted. Furthermore, vancomycin‐resistant isolates of enterococci, mainly vancomycin‐resistant *E. faecium* pose a growing challenge in the hospital setting and are frequently managed with alternative antimicrobial therapies, primarily linezolid or daptomycin [11, 12].

The aim of this retrospective study was to evaluate the clinical outcomes of any penicillin‐based therapies (including ampicillin, ampicillin/sulbactam, or piperacillin/tazobactam as mono‐ or combination therapy) compared with alternative nonpenicillin‐based enterococcal‐active therapies (glycopeptides, linezolid, or daptomycin) in hospitalized adult patients with vancomycin‐susceptible enterococcal BSI.

## 2. Patients and Methods

### 2.1. Study Population and Design

This retrospective monocenter cohort study was conducted at Jena University Hospital, a 1400‐bed academic hospital in Thuringia, Germany [13]. The study protocol received approval from the local ethics committee of the Friedrich Schiller University of Jena (Approval Number 2019‐1598). Due to the retrospective design of the study, the requirement for informed consent was waived. The research adhered to the Declaration of Helsinki and national standards.

The study screened adult patients (≥18 years) with at least one positive blood culture (BC) for vancomycin‐susceptible *Enterococcus species* (minimum inhibitory concentration of vancomycin: ≤4 mg/L; http://www.eucast.org) between January 1, 2017 and December 31, 2018. Inclusion criteria required patients with enterococcal BSI who received adequate enterococcal‐active therapy (ampicillin, ampicillin/sulbactam, piperacillin/tazobactam, glycopeptides, linezolid, or daptomycin). Patients were excluded if they had a treatment duration of less than 48 h, received inadequate therapy (in vitro resistance or tigecycline monotherapy), or had missing data on antiinfective therapy or outcomes.

### 2.2. Microbiological Diagnostics

BCs were collected as part of routine diagnostics, with one to three BC sets consisting of one aerobic and one anaerobic bottle being drawn per sampling. BC bottles were then incubated at 35°C using the BACTEC FX system (BD Diagnostics, Heidelberg, Germany), as previously described [14]. Positive BCs were microscopically examined using Gram staining and subcultured on Columbia blood and chocolate agar. Bacterial isolates were identified by VITEK MS MALDI‐TOF or VITEK 2 (bioMérieux, Marcy‐l’Étoile, France). Antimicrobial susceptibility testing was performed in accordance with EUCAST criteria using VITEK 2 for aminopenicillins, glycopeptides, and linezolid. MIC testing for daptomycin was performed using gradient diffusion strips (MTS Daptomycin, liofilchem, Roseto degli Abruzzi, Italy). To evaluate in vitro susceptibility to piperacillin/tazobactam in ampicillin‐susceptible isolates, rapid susceptibility testing results from disk diffusion performed directly from positive BCs were taken into account, with inhibition zone diameters ≥ 22 mm cautiously interpreted as susceptible [15], with no EUCAST breakpoints available at the time of analysis.

### 2.3. Data Management

The following data were extracted from the patients’ electronic medical records: age, sex, body mass index (BMI), comorbidities, Charlson comorbidity index, implanted devices (cardiac, orthopedic), source of enterococcal BSI (no identified source, abdominal, urinary, vascular catheter‐related, surgical site, skin/soft tissue, and endocarditis), infection origin (hospital‐acquired versus community‐acquired), admission to an intensive care unit (ICU) and requirement of catecholamines, date of positive BCs with *Enterococcus* species, in vitro susceptibility results, presence of polymicrobial bacteremia, active agents and class of antibiotics used for targeted treatment, duration and dosages of antibiotic therapy, source control management, potential antibiotic‐related adverse events (bone marrow toxicity, a >5‐fold increase in creatine kinase level, a >2‐fold rise in creatinine level, eosinophilia >20%, or *Clostridioides difficile* infection), length of hospital stay (LOS), and death within the first 90 days after positive BC. Data were documented in a pseudonymized manner using a password‐protected file. Random checks were carried out to ensure data accuracy and completeness.

### 2.4. Outcome Analyses

The study’s primary outcome was the 30‐day mortality following the onset of enterococcal BSI. Secondary outcomes were 90‐day mortality, recurrent bacteremia, LOS, and potential antibiotic‐related adverse events. Relapse BSI was defined as the occurrence of microbiologically confirmed enterococcal bacteremia after the completion of antimicrobial treatment [11, 16].

### 2.5. Statistical Analysis

Data analysis was performed using *SPSS software*, Version 30.0 (SPSS Inc., Chicago, IL, USA) and *R* Version 4.5.0 (R Core Team, 2025; R Foundation for Statistical Computing, Vienna, Austria), using the *DescTools* (Signorell, 2025), *tidyverse* (Wickham, 2019), *Matching* (Sekhon, 2011), survey (Lumley, 2024), and *ggpubr* (Kassambara, 2023) packages. The demographics, comorbidities, disease severities, and BSI sources of the patients were compared across three different treatment regimens: penicillin‐based therapy, alternative therapy, and combined penicillin/alternative therapy. Categorical variables were presented as frequencies and percentages, while continuous variables were expressed as medians with interquartile ranges (Q1 and Q3). Baseline characteristics were compared among the three treatment groups using Fisher exact tests for nominal data and the Kruskal–Wallis test for numeric data. Fisher exact tests, with a two‐sided *p*‐value threshold of <0.05, were also employed to compare the primary outcome, 30‐day mortality, and secondary outcomes, including 90‐day mortality, relapse BSI, and potential antibiotic‐related events, between patients receiving any penicillin‐based therapy and those receiving alternative therapy. Additionally, subgroup analyses were performed for patients with *E. faecalis* monoinfection, *E. faecium* mononinfection, and patients with leukemia or lymphoma. Logistic regression analyses were performed to identify potential predictors of 30‐day mortality. Variables included in the multivariable logistic regression analysis were age, community‐acquired infection, polymicrobial bacteremia, *E. faecium* monoinfection, leukemia or lymphoma, septic shock, intra‐abdominal infection, urinary tract infection, any penicillin‐based regimen (including penicillin/alternative therapy), any glycopeptide‐based regimen, any linezolid‐based regimen, dual enterococcal combination therapy, any antibiotic combination therapy, and early source control. Since ampicillin susceptibility is a strong predictor for a penicillin‐based regimen, we estimated propensity scores using parametric logistic regression with any penicillin‐based regimen as the outcome and ampicillin susceptibility as the predictor. Weights were calculated using two methods, inverse probability of treatment weighting (IPTW) and matching weights (MWs) [17]. We used absolute standardized mean differences (ASMDs) for the assessment of covariate balance across treatment groups on unweighted and weighted data (Figure S1). Since the covariate balance of IPTW data was insufficient (ASMD > 0.1), MWs were used in the weighted analysis. A two‐tailed *p*‐value  < 0.05 was considered significant for the multivariable model.

## 3. Results

### 3.1. Characteristics of the Study Population

**Figure 1:**
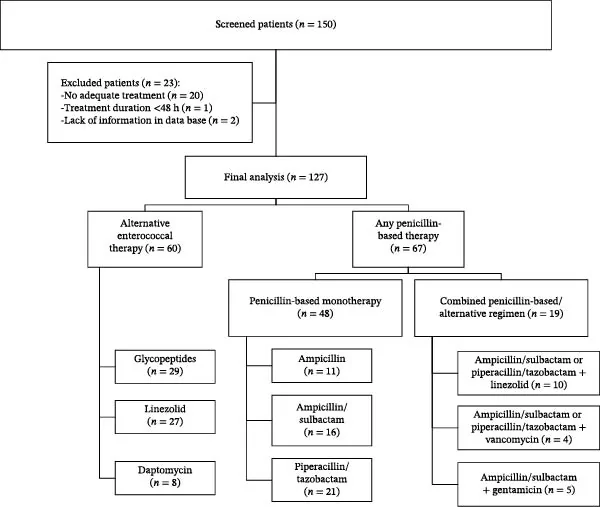
Flow chart of the study population.

A total of 150 adult patients with enterococcal BSI were initially screened. Of these, 23 patients were excluded due to inadequate treatment (*n* = 20), a treatment duration of less than 48 h (*n* = 1), and missing data (*n* = 2). Thus, 127 patients (85.3%) were included in the final analysis (refer to Figure 1). The median age of the study cohort was 73.0 years, with a predominance of male patients (*n* = 86, 67.7%). At the onset of BSI, 52 patients (40.9%) were receiving treatment in an ICU, and 36 patients (28.3%) required catecholamines due to septic shock. As shown in Table 1, the most prevalent comorbidities among the study population were solid tumor diseases (*n* = 43, 33.9%) and heart diseases (*n* = 41, 32.3%).

**Table 1:** Demographics, source of infection, and presence of septic shock—overall and stratified for the three therapy groups (penicillin‐based monotherapy, alternative therapy, and combined penicillin‐based/alternative regimen).

Variable	All study patients (*n* = 127)	Penicillin‐based monotherapy (*n* = 48)	Alternative enterococcal therapy (*n* = 60)	Combined penicillin‐based regimen/alternative enterococcal therapy (*n* = 19)	Univariate *p*‐value
Baseline parameters					
Male Female	86 (67.7) 41 (32.3)	33 (68.8) 15 (31.2)	40 (66.7) 20 (33.3)	13 (68.4) 6 (31.6)	0.966
Age in years (median, IQR)	73.0 (64.0, 79.0)	76.5 (69.5, 81.5)	66 (61.5, 76.5)	74 (66, 80)	**<0.001**
BMI (median, IQR)	27.1 (24.2, 30.9)	26.5 (22.0, 32.0)	27.0 (24.5, 30.6)	28.7 (26.4, 33.7)	0.235
Community‐acquired infection	41 (32.3)	28 (58.3)	9 (15.0)	4 (21.1)	**<0.001**
Enterococcal species and polymicrobial bacteremia					
Ampicillin‐susceptible *Enterococcus* sp.^a^	75 (59.1)	48 (100)	8 (13.3)	19 (100)	**<0.001**
*E. faecalis* monoinfection	52 (40.9)	32 (66.7)	5 (8.3)	15 (78.9)	**<0.001**
*E. faecium* monoinfection	49 (38.6)	1 (2.1)	47 (78.3)	1 (5.3)	**<0.001**
Other enterococcal species^b^	4 (3.1)	2 (4.2)	2 (3.3)	0 (0)	1.0
Polymicrobial bacteremia	24 (18.9)	15 (31.2)	6 (10.0)	3 (15.8)	**0.018**
Comorbidities					
Charlson comorbidity score	7.0 (5.0, 9.0)	7 (5.0, 10.0)	6.5 (5.0, 10.0)	5.0 (4.0, 8.0)	0.356
Heart disease^c^	41 (32.3)	16 (33.3)	19 (31.7)	6 (31.6)	1.0
Peripherial vascular disease	17 (13.4)	7 (14.6)	7 (11.7)	3 (15.8)	0.824
Cerebrovascular disease	26 (20.5)	15 (31.2)	7 (11.7)	4 (21.1)	**0.044**
Lung disease	24 (18.9)	10 (20.8)	13 (21.7)	1 (5.3)	0.254
Liver disease	13 (10.2)	4 (8.3)	8 (13.3)	1 (5.3)	0.668
Kidney disease	31 (24.4)	8 (16.7)	17 (28.3)	6 (31.6)	0.247
Solid tumor disease	43 (33.9)	16 (33.3)	23 (38.3)	4 (21.1)	0.095
Leukemia or lymphoma	16 (12.6)	1 (2.0)	13 (21.7)	2 (10.5)	**0.006**
Intracardial device^d^	18/125 (14.4)	7/47 (14.9)	6/60 (10.0)	5/18 (27.8)	0.188
Orthopedic device	13 (10.2)	8 (16.7)	3 (5.0)	2 (10.5)	0.119
Source of infection, metastatic infections, septic shock, and treatment duration					
Primary bacteremia	27 (21.3)	9 (18.8)	13 (21.7)	5 (26.3)	0.765
Vascular catheter‐related	13 (10.2)	5 (10.4)	6 (10.0)	2 (10.5)	1.000
Urinary tract infection	27 (21.3)	16 (33.3)	9 (15.0)	2 (10.5)	**0.042**
Intra‐abdominal infection	42 (33.1)	10 (20.8)	27 (45.0)	5 (26.3)	**0.025**
Skin/soft tissue infection	7 (5.5)	3 (6.2)	3 (5.0)	1 (5.3)	1.000
Surgical site infection	7 (5.5)	4 (8.3)	3 (5.0)	0 (0)	0.663
More than one clinical focus	16 (12.6)	5 (10.4)	9 (15.0)	2 (10.5)	0.818
Early source control within 72 h	46 (36.2)	21 (43.8)	20 (33.3)	5 (26.3)	0.374
Metastatic infections					
‐Endocarditis	13 (10.2)	4 (8.3)	4 (6.7)	5 (26.3)	0.063
‐Osteomyelitis/spondylodiscitis	5 (3.9)	3 (6.2)	1 (1.7)	1 (5.3)	0.364
ICU admission	52 (40.9)	13 (27.1)	30 (50)	9 (47.4)	**0.045**
Septic shock	36 (28.3)	7 (14.6)	23 (38.3)	6 (31.6)	**0.019**
Treatment duration (median, IQR)	11 (8, 17)	11 (8, 14)	13.5 (8, 17)	11 (9, 20)	0.360

*Note*: Significant *p*‐values < 0.05 are shown in bold.

^a^Ampicillin‐susceptible *Enterococcus* species included *E. faecalis* (*n* = 68), *E. faecium* (*n* = 5), *E. hirae* (*n* = 1), and *E. avium* (*n* = 2).

^b^Other enterococcal species included *E. avium* (*n = 2*), *E. raffinosus* (*n = 1*), and *E. hirae* (*n = 1*).

^c^Heart disease included congestive heart failure, coronary heart disease, and St.p. myocardial infarction.

^d^Intracardial device included valve replacement, implanted pacemakers, and ICDs.

The primary pathogen identified was *E. faecalis*, accounting for 68 cases (52 cases of monomicrobial BSI and 16 cases of polymicrobial BSI), followed by *E. faecium* with 57 cases (49 cases of monomicrobial BSI and 8 cases of polymicrobial BSI). Additional enterococcal species detected in BCs included *E. avium* (2 cases), *E. raffinosus* (1 case), and *E. hirae* (1 case). Concurrent infections with two different *Enterococcus* species were observed in three patients: *E. faecalis* and *E. faecium* in two cases and *E. faecalis* and *E. avium* in one case. Among the enterococcal species, those susceptible to aminopenicillin—specifically *E. faecalis* (*n* = 68), *E. faecium* (*n* = 5), *E. hirae* (*n* = 1), and *E. avium* (*n* = 2)—comprised 59.1% of all enterococcal BSI cases. The predominant source of BSIs was intra‐abdominal infections (*n* = 42, 33.1%), followed by urinary tract infections (*n* = 27, 21.3%), vascular catheter‐related infections or endocarditis (each: *n* = 13, 10.2%), skin/soft tissue infections or surgical site infections (each: *n* = 7, 5.5%), and spondylodiscitis (*n* = 5, 3.9%). Sixteen patients (12.6%) presented with more than one clinical source of infection. The median treatment duration was 11 days (interquartile range: 8–17 days).

In the final analysis of 127 patients, 67 patients (52.1%) were definitively treated with any penicillin‐based regimen, 48 individuals (37.8%) received a penicillin‐based monotherapy, and 19 individuals (14.9%) received a combination therapy. Penicillin‐based monotherapy included ampicillin (11 patients), ampicillin/sulbactam (16 patients), and piperacillin/tazobactam (21 patients). The penicillin‐based combined therapies included a penicillin derivative plus either linezolid (*n* = 10), vancomycin (*n* = 4), or gentamicin (*n* = 5).

A total of 60 patients (47.2%) received alternative nonpenicillin‐based regimens, consisting of various glycopeptides (29 patients), linezolid (27 patients), or daptomycin (8 patients). Among these 60 patients, 7 patients received combinations of two different alternative regimens (daptomycin/gentamicin *n* = 1, glycopeptide/daptomycin *n* = 1, glycopeptide/linezolid *n* = 2, linezolid/gentamicin *n* = 1, glycopeptide/tigecycline *n* = 1, and glycopeptide/gentamicin *n* = 1).

Table 1 provides a comparative analysis of demographic distribution, *Enterococcal species*, comorbidities, sources of infection, and the incidence of septic shock across three treatment groups: penicillin‐based monotherapy, an alternative regimen, and a combined penicillin‐based/alternative regimen. Notable differences were observed in baseline characteristics among the treatment groups. Patients with community‐acquired infections, *E. faecalis* monoinfection, polymicrobial bacteremia, or urinary tract infections were more frequently treated with penicillin‐based monotherapies. In contrast, *E. faecium* monoinfection, hematological malignancies, intra‐abdominal infections, or septic shock were associated with increased utilization of alternative treatment regimens.

### 3.2. Primary Outcome

Detailed results of the primary outcome are presented in Table 2. A total of 33 patients (26.0%) died within 30 days after the onset of BSI. Patients treated with any penicillin‐based therapy had a lower 30‐day mortality rate of 13.4% than those treated with an alternative nonpenicillin‐based therapy, who experienced a mortality rate of 40.0% (*p* = 0.001). In subgroup analyses, the 30‐day mortality rates for patients with *E. faecalis* monoinfection or patients with leukemia or lymphoma were low, with 13.5% and 12.5%, while patients with *E. faecium* monoinfection had a high 30‐day mortality rate of 42.9%.

**Table 2:** Primary and secondary outcomes of any penicillin‐based therapy versus alternative treatment in patients with enterococcal bloodstream infections and stratified for patients with *E. faecalis* or *E. faecium* monoinfection.

Outcome parameters	All study patients (*n* = 127)	Any penicillin‐based therapy (*n* = 67)	Alternative enterococcal therapy (*n* = 60)	Univariate *p*‐value
30‐day mortality	33 (26.0)	9 (13.4)	24 (40.0)	0.001
‐*E. faecalis* monoinfection (*n* = 52)	7/52 (13.5)	5/47 (10.6)	2/5 (40.0)	—
‐*E. faecium* monoinfection (*n* = 49)	21/49 (42.9)	0/2 (0)	21/47 (44.7)	—
‐Leukemia or lymphoma (*n* = 16)	2/16 (12.5)	0/3 (0)	2/13 (15.4)	—
90‐day mortality	34 (26.8)	9 (13.4)	25 (41.7)	<0.001
‐*E. faecalis* monoinfection (*n* = 52)	7/52 (13.5)	5/47 (10.6)	2/5 (40.0)	—
‐*E. faecium* monoinfection (*n* = 49)	22/49 (44.9)	0/2 (0)	22/47 (46.8)	—
‐Leukemia or lymphoma (*n* = 16)	2/16 (12.5)	0/3 (0)	2/13 (15.4)	—
Relapse BSI	8 (6.3)	3 (4.5)	5 (8.3)	0.475
‐*E. faecalis* monoinfection (*n* = 52)	3/52 (5.8)	2/47 (4.3)	1/5 (20.0)	—
‐*E. faecium* monoinfection (*n* = 49)	4/49 (8.2)	0/2 (0)	4/47 (8.5)	—
‐Leukemia or lymphoma (*n* = 16)	3/16 (18.8)	0/3 (0)	3/13 (23.1)	—
Length of stay in days (median, IQR)	28 (14; 48)	21 (12; 35)	35 (22.5; 53)	<0.001
‐*E. faecalis* monoinfection (*n* = 52)	23 (12.3; 33.8)	23 (13; 33)	28 (10; 52.5)	—
‐*E. faecium* monoinfection (*n* = 49)	36 (25.5; 53)	38.5 (34;–)	36 (25; 53)	—
‐Leukemia or lymphoma (*n* = 16)	52.5 (33.3; 57.3)	37 (11;–)	53 (34.5; 56.5)	—
Potential antibiotic‐related adverse events				
>twofold rise in creatinine	10/121 (8.3)	4/66 (6.1)	6/55 (10.9)	0.509
>fivefold rise in CK	7/82 (8.5)	2/43 (4.7)	5/39 (12.8)	0.249
Bone marrow toxicity	38/127 (29.9)	17/67 (25.4)	21/60 (35.0)	0.251
Eosinophilia > 20%	1/59 (1.7)	0/30 (0)	1/29 (3.4)	0.492
Clostridioides difficile infection	2/127 (1.6)	0/67 (0)	2/60 (3.3)	0.221

### 3.3. Secondary Outcomes

In our cohort, 34 patients (26.8%) died within 90 days. As shown in Table 2, 90‐day mortality rates were lower in patients with any penicillin‐based therapy (13.4%) or *E. faecalis* monoinfection (13.5%) compared to those in patients with alternative therapy (41.7%) or *E. faecium* monoinfection (44.9%). In terms of LOS, patients on alternative regimens had a significantly longer median length of stay of 35 days (IQR, 22.5–53.0) compared to those treated with a penicillin‐based regimen, who had a median stay of 21 days (IQR, 12–35 days) (*p*  < 0.001). There were no significant differences in the rates of relapse BSI or potential adverse events between any penicillin‐based and alternative therapy groups (*p*  > 0.05 for all tests). Subgroup analyses showed that patients with leukemia or lymphoma who received nonpenicillin‐based alternative therapy exhibited the highest relapse BSI rates (23.1%) and had an extended length of inpatient stays (median 53 days).

### 3.4. Predictors of 30‐Day Mortality

**Figure 2:**
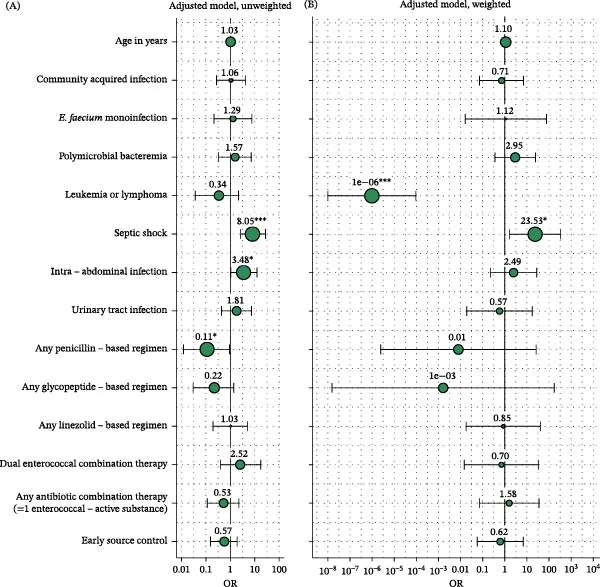
Forest plot for (A) multivariable logistic regression analysis and (B) for weighted multivariable logistic regression analysis of predictors of 30‐day overall mortality in patients with vancomycin‐susceptible enterococcal bloodstream infection. The model includes age, community‐acquired infection, *E. faecium* monoinfection, polymicrobial bacteremia, presence of leukemia or lymphoma, septic shock, source of bloodstream infection (intra‐abdominal infection and urinary tract), antibiotic regimen (penicillin‐based, glycopeptide‐based, linezolid‐based, dual enterococcal combination, or any combination), and early source control. An asterisk ( ^∗^) represents a *p*‐value  < 0.05, two asterisks ( ^∗∗^) represent a *p*‐value  < 0.01, and three asterisks ( ^∗∗∗^) represent a *p*‐value  < 0.001.

Unweighted and weighted multivariable regression analyses are shown in Table 3. In unweighted analysis, an association with 30‐day mortality was found for septic shock, intra‐abdominal infection, and penicillin‐based therapy. After weighting for ampicillin susceptibility, only septic shock (adjusted OR [aOR] with 95% CI: 23.53 [1.66; 334.54]) was identified as an independent positive predictor (see also Figure 2). The presence of leukemia or lymphoma (aOR with 95% CI: 1.0e–06 [1.0e–08; 9.5e–05]) was a negative predictor of 30‐day mortality; however, only 16 patients with hematologic malignancies were included in the study.

**Table 3:** Independent predictors of 30‐day all‐cause mortality in patients with vancomycin‐susceptible enterococcal bloodstream infection.

Variable	Early death within 30 days		Unweighted model		Weighted model	
	No (*N* = 94)	Yes (*N* = 33)	Odds ratio (95% CI)	Adjusted odds ratio (95% CI)	Odds ratio (95% CI)	Adjusted odds ratio (95% CI)
Age in years (median, IQR)	74 (64–78)	69 (61.5–81)	0.99 (0.96–1.03)	1.03 (0.98–1.09)	1.00 (0.93–1.08)	1.10 (0.99–1.22)
Community‐acquired infection	32 (34.0%)	9 (27.3%)	0.73 (0.29–1.70)	1.06 (0.27–4.23)	0.83 (0.12–5.56)	0.71 (0.07–7.02)
*E. faecium* monoinfection	28 (29.8%)	21 (63.6%)	**4.13 (1.82–9.76)**	1.29 (0.22–7.62)	2.81 (0.18–44.58)	1.12 (0.02–77.15)
Polymicrobial bacteremia	20 (21.3%)	4 (12.1%)	0.51 (0.14–1.49)	1.57 (0.32–7.22)	0.81 (0.17–3.86)	2.95 (0.36–24.42)
Leukemia or lymphoma	14 (14.9%)	2 (6.1%)	0.37 (0.056–1.422)	0.34 (0.036–2.152)	**9.7e–08 (1.7e–08–5.7e–07)**	**1.0e–06 (1.0e–08–9.5e–05)**
Septic shock	16 (17.0%)	20 (60.6%)	**7.50 (3.16–18.61)**	**8.05 (2.64–27.46)**	**20.18 (2.87–141.95)**	**23.53 (1.66–334.54)**
Intra‐abdominal infection	24 (25.5%)	18 (54.5%)	**3.50 (1.54–8.12)**	**3.48 (1.04–12.27)**	2.32 (0.34–16.02)	2.49 (0.22–27.84)
Urinary tract infection	21 (22.3%)	6 (18.2%)	0.77 (0.26–2.03)	1.81 (0.43–7.38)	2.29 (0.30–17.54)	0.57 (0.02–17.36)
Any penicillin‐based regimen	58 (61.7%)	9 (27.3%)	**0.23 (0.09–0.54)**	**0.11 (0.01–0.92)**	0.26 (0.05–1.30)	0.01 (2.4e–06 −26.09)
Any glycopeptide‐based regimen	27 (28.7%)	6 (18.2%)	0.55 (0.19–1.41)	0.22 (0.03–1.37)	**0.04 (3.0e–03 −0.37)**	1.0e–03 (1.5e–08 −173.02)
Any linezolid‐based regimen	20 (21.3%)	17 (51.5%)	**3.93 (1.70–9.26)**	1.03 (0.20–5.09)	**32.97 (7.13–152.54)**	0.85 (0.02–40.75)
Dual enterococcal combination therapy	20 (21.3%)	6 (18.2%)	0.82 (0.28–2.17)	2.52 (0.39–17.71)	0.30 (0.05–1.68)	0.70 (0.02–33.79)
Any antibiotic combination therapy (at least one enterococcal‐active substance)	39 (41.5%)	16 (48.5%)	1.33 (0.60–2.96)	0.53 (0.11–2.23)	1.67 (0.25–11.08)	1.58 (0.07–35.45)
Early source control	32 (34.0%)	14 (42.4%)	1.43 (0.63–3.21)	0.57 (0.15–1.90)	**10.23 (2.20–47.59)**	0.62 (0.06–6.80)

*Note*: Significant *p*‐values < 0.05 are shown in bold.

## 4. Discussion

This real‐world study investigated clinical outcomes, in particular 30‐day mortality, in hospitalized adult patients with enterococcal BSI caused by vancomycin‐susceptible isolates, with a focus on penicillin‐based regimens. Ampicillin‐, ampicillin/sulbactam‐, or piperacillin/tazobactam‐based therapies were used to definitively treat enterococcal BSI in 52.8% of cases at our university hospital. *E. faecalis* emerged as the predominant BSI pathogen, and 59.1% of all BSI pathogens were susceptible to aminopenicillins. Nevertheless, 10.7% of patients with ampicillin‐susceptible enterococcal BSI received alternative definitive therapies, mainly glycopeptides and linezolid. In contrast, recent retrospective studies from the United States of America and Korea reported a higher prevalence of nonbeta‐lactam agents in cases of ampicillin‐susceptible enterococcal BSI, with rates of 27% and 56%, respectively [18, 19]. A single‐center study from New Hampshire, USA, identified several factors associated with the use of nonbeta‐lactam therapies in ampicillin‐susceptible BSI, including younger age, any penicillin allergy, history of cancer, end‐stage renal disease, and polymicrobial bacteremia [18].

Notably, the present study included a significant proportion of severely ill patients, as 40.9% of patients were admitted to the ICU, and 28.3% of patients experienced septic shock. One in four patients died within 30 days, a mortality rate consistent with previous research [2, 20, 21].

In comparing clinical outcomes, we found that patients receiving nonpenicillin‐based therapies or those with *E. faecium* monoinfection exhibited higher mortality rates (40%–45%) and an extended LOS (median 35–36 days). Similarly, in a recent study from Korea, glycopeptide treatment in ampicillin‐susceptible *E. faecalis* or *E. faecium* bacteremia had 3.7‐fold higher odds of 28‐day mortality than with treatment with an ampicillin‐containing regimen [19]. Additionally, previous studies have shown that BSIs by *E. faecium* are associated with higher 30‐day in‐hospital mortality rates compared to those by *E. faecalis* [5, 21, 22], despite *E. faecalis* generally being considered more virulent due to higher prevalence of classical virulence factors and strong biofilm‐forming capacity [23, 24]. On the other hand, *E. faecium* is better adapted to the nosocomial environment and has a high ability to acquire and disseminate antibiotic resistance [25, 26]. In a retrospective population‐based surveillance study of the Calgary Health zone, Canada, encompassing 710 episodes of enterococcal BSI from 2000 to 2008, *E. faecalis* was associated with reduced 30‐day mortality (OR: 0.44, 95% CI: 0.30–0.67), whereas the Charlson severity index (OR: 1.33, 95% CI: 1.2–1.4) and nosocomial infection (OR: 2.15, 95% CI: 1.41–3.27) were linked to increased 30‐day mortality [5]. Similarly, a retrospective study from Auckland City Hospital, New Zealand, involving 212 patients with vancomycin‐susceptible *E. faecalis* or *E. faecium* bacteremia, found that 30‐day mortality was significantly associated (*p*  < 0.05) with cirrhosis, malignancy, *E. faecium* bacteremia and the absence of active antimicrobial therapy [21]. In contrast, a recent prospective multicenter cohort study from Spain that included 307 patients with monomicrobial enterococcal BSI (186 due to *E. faecalis* and 121 due to *E. faecium*) was also unable to show a difference in in‐hospital mortality rates between patients with *E. faecium* BSI (21.5%) and *E. faecalis* BSI (20.4%) [6].

Nevertheless, *E. faecium* acted as a strong confounder in the present study, effectively dividing our cohort into two distinct groups: patients without *E. faecium* receiving penicillin antibiotics and those with *E. faecium* not receiving penicillin derivatives. Only two patients with *E. faecium* had penicillin‐based therapy, and only five patients with *E. faecalis* had no penicillin‐based therapy. However, after propensity score weighting for aminopenicillin susceptibility, neither the choice of treatment nor the *Enterococcus* species were identified as independent predictors of 30‐day mortality in the multivariable logistic regression analysis. Septic shock emerged as the sole positive predictor of 30‐day mortality. Conversely, leukemia or lymphoma was identified as the only negative predictor of 30‐day mortality in our study. Patients with leukemia and lymphoma demonstrated a low mortality rate of 12.5% (2/16 patients), despite a high prevalence of *E. faecium* monoinfection (10/16, 62.5%). Given the limited number of patients with hematologic malignancies included in the study, the results may be influenced by sampling variability and should therefore be interpreted with caution. Although mortality was lower in this subgroup, these patients experienced significant rates of enterococcal relapse BSI (3/16, 18.8%) and prolonged LOS (median 52.5 days), even in the absence of reported metastatic infections, such as endocarditis or osteomyelitis. These outcomes may partially reflect a higher incidence of vascular catheter‐related BSI (4/16, 25%) compared to those without hematologic malignancies (9/111, 8.1%) and insufficient early source control. Notably, only 1 out of 16 patients (6.3%) with an underlying hematologic malignancy received early source control, in contrast to 45 out of 111 patients (40.5%) without such conditions. Antimicrobial stewardship interventions in hematology–oncology units, in particular, pose significant challenges.

Other retrospective cohort studies from Birmingham, USA, or Lausanne University Hospital, Switzerland, involving adult patients with enterococcal BSIs, also demonstrated that not only appropriate antiinfective treatment but also early consultation of an infectious diseases specialist were associated with a significant reduction of 30‐day in‐hospital mortality [22, 27].

Our study has several limitations. First, this was a single‐center observational study; the sample size was limited, and the choice, dosage, and duration of treatment were at the discretion of the treating physicians and were not standardized; therefore, the generalizability of our findings may be limited. Second, due to the retrospective study design, all collected patient data were recorded only from the electronic patient files, so we had no control over the quality and accuracy of the documented information. Third, we did not assess the Pitt bacteremia score, a widely recognized severity‐of‐illness score for estimating early mortality, particularly in patients with Gram‐negative BSIs [14, 28] and patients with nonbacteremic carbapenem‐resistant Enterobacteriaceae infections [29]. Fourth, we cannot determine whether repeated BSI episodes were true relapse events or new infections as isolates were not available for molecular typing.

In conclusion, our study indicates that penicillin‐based therapy remains an effective and safe treatment option for patients with enterococcal BSI. However, after adjusting for aminopenicillin susceptibility using propensity score weighting, we did not demonstrate an association with improved survival. Although our study was not designed to evaluate the World Health Organization (WHO) AWaRe framework for optimizing antibiotic use and mitigating resistance [30], our findings are compatible with current antimicrobial stewardship principles that favor the use of narrower‐spectrum Access antibiotics, when clinically appropriate and supported by susceptibility testing.

## Author Contributions

Christina Bahrs, Stefan Hagel, and Mathias W. Pletz contributed to the study design. Stefan Glöckner collected microbiological data. Thalia Landrock and Christina Bahrs collected the clinical data. Christina Bahrs supervised the study. All authors were involved in critical revision of the manuscript and additional important intellectual content and data interpretation.

## Funding

This study was financed by internal funding.

## Disclosure

Christina Bahrs, Thalia Landrock, and Lisa Schneider had full access to all the data in the study, took responsibility for the integrity of the data and the accuracy of the data analysis, performed the data analysis, and drafted the manuscript.

## Ethics Statement

The authors confirm that the ethical policies of the journal, as noted on the journal’s author guideline page, have been adhered to and the appropriate ethical review committee approval has been received by the local ethics committee of the Friedrich Schiller University Jena (Ethical Approval Number 2019‐1598). The research was conducted in accordance with the Declaration of Helsinki and national and institutional standards.

## Conflicts of Interest

The authors declare no conflicts of interest.

## Supporting Information

Additional supporting information can be found online in the Supporting Information section.

## Supporting information


**Supporting Information** The supporting information includes Figure S1 that shows unweighted inverse probability (IPTW) and match weighted (MW) absolute standardized mean differences (ASMDs) of ampicillin‐susceptible enterococcal strains between penicillin‐based therapy regimens and other therapies. Since covariate balance of IPTW data was insufficient (ASMD > 0.1), MW were used in the weighted.

## Data Availability

The datasets used and/or analyzed during the current study are available from the corresponding author upon reasonable request.
