# Supplementary material for: Clinical Outcomes of Penicillin‐Based Therapy Compared to Alternative Enterococcal Treatment in Patients With Vancomycin‐Susceptible Enterococcal Bloodstream Infections: A Monocentric Cohort Study
Source: Transbound Emerg Dis. 2026 Aug 27;2026:3822590. doi: 10.1155/tbed/3822590 (PMC13522629; doi:10.1155/tbed/3822590)
Supplement: Supplementary file 1 [file TBED-2026-3822590-s001.docx]

Figure S1 - Unweighted inverse probability (IPTW)and match weighted (MW) absolute standardized mean differences of ampicillin susceptible enterococcal strains between penicillin-based therapy regimens and other therapy.
